# Trends in Reported Assisted Living Facility Acquisitions by Private Equity Firms From 2006-2023

**DOI:** 10.1093/geroni/igaf122.1407

**Published:** 2025-12-31

**Authors:** Jennifer Bunker, Gauri Gadkari, Yashaswini Singh, John Bowblis, Sean Huang, Lindsey Smith, Momotazur Rahman, Kali Thomas

**Affiliations:** Johns Hopkins University, Baltimore, Maryland, United States; Brown University, Providence, Rhode Island, United States; Brown University School of Public Health, Providence, Rhode Island, United States; Miami University, Oxford, Ohio, United States; Georgetown University, Washington, District of Columbia, United States; Oregon Health & Science University, Portland, Oregon, United States; Brown University, Providence, Rhode Island, United States; Johns Hopkins University, Baltimore, Maryland, United States

## Abstract

Assisted living (AL) facilities provide a supportive alternative to nursing homes for approximately 1 million older adults across 34,000 U.S. facilities. While private equity (PE) investment in nursing homes has grown, its impact on AL remains unclear. This research examines PE acquisitions of ALs from 2006-2023, analyzing transaction volume, value, and geographic trends. Using LevinAssociates databases amended with SEC reports, Pitchbook, S&P NetAdvantage, press releases, and company websites, we identified 254 PE transactions involving 711 AL facilities. Transactions ranged from 1 to 134 ALs, with 70.9% (n = 180) involving a single facility. ALs were located in 46 states, with Florida (n = 69), California (n = 62), and Texas (n = 46) comprising 25% of acquisitions. Transaction value was reported for 66.9% (n = 170) of deals (n = 558 ALs), ranging from $750,000 to $1.8 billion (median $29.1M, mean $79.6M). Average value per facility ranged from $750,000 to $95.5M (median $22.5M, mean $25.6M). Reported transactions increased over time, peaking at 35 in 2021, with 65.7% occurring between 2015-2021. AL acquisitions grew annually, from two in 2008 to a peak 184 in 2015. Reported PE investment in AL has risen significantly in the past decade. This study provides a novel dataset to understand PE trends in AL, offering insights into its growing role in this important sector of long-term care.

